# Scanning Electron Microscopy Reveals the Antennal Micromorphology of *Lamprodila* (*Palmar*) *festiva* (Coleoptera: Buprestidae), an Invasive Pest of Ornamental Cupressaceae in Western Palaearctic

**DOI:** 10.3390/biology9110375

**Published:** 2020-11-04

**Authors:** Michel J. Faucheux, Tamás Németh, Johana Hoffmannova, Robin Kundrata

**Affiliations:** 1 Laboratoire d’Endocrinologie des Insectes Sociaux, Faculté des Sciences et des Techniques, 2 rue de la Houssinière, B.P. 92208, F-44322 Nantes CEDEX 03, France; faucheux.michel@free.fr; 2 Department of Zoology, Hungarian Natural History Museum, Baross utca 13, H-1088 Budapest, Hungary; haesito@gmail.com; 3 Department of Zoology, Faculty of Science, Palacky University, 17. listopadu 50, CZ-771 46 Olomouc, Czech Republic; johana.hoffmannova01@upol.cz

**Keywords:** antennal sensilla, Cypress borer, jewel beetle, sexual dimorphism, sensory fields, sensory complex

## Abstract

**Simple Summary:**

The jewel-beetles, Buprestidae, comprise some economically important invasive pest species. The Cypress jewel beetle, *Lamprodila* (*Palmar*) *festiva* *festiva* (Linnaeus, 1767), is a new invasive pest of ornamental Cupressaceae, which has recently expanded its range from the Mediterranean region northwards to central and eastern Europe, and to the Russian Black Sea coast. In this study, we used scanning electron microscopy to examine the morphology, numbers, distribution and possible functions of antennal sensilla in both sexes of *L. festiva*. In total, we identified 15 different (sub)types of sensilla, of which two are present only in females. We discuss possible functions of all examined sensilla and compare them with those in other Buprestidae or other insects. Our study should serve as background information for subsequent chemical ecology research focused mainly on the olfactory sensory system of this rapidly spreading invasive pest.

**Abstract:**

The Cypress jewel beetle, *Lamprodila* (*Palmar*) *festiva festiva* (Linnaeus, 1767), is a serious invasive pest of ornamental Cupressaceae, which has recently expanded its range from the Mediterranean region northwards to central and eastern Europe, and to the Russian Black Sea coast. In this study, we conducted a scanning electron microscopy study of the micromorphology of the male and female antennae of *L. festiva* to examine the morphology, numbers, distribution, and possible functions of antennal sensilla. Most sensilla are located in the sensory fields within the apical depressions on antennomeres IV–XI. We identified four main types of antennal sensilla in *L. festiva*: sensilla chaetica (seven subtypes, of which two occur only in females), sensilla basiconica (five subtypes), multiporous grooved pegs (two subtypes), and Böhm sensilla. Females have relatively more sensilla chaetica and multiporous grooved pegs, whereas males have more sensilla basiconica. We discuss possible functions of all examined sensilla and compare them with those in other Buprestidae or other insects. Our study should serve as background information for advanced electrophysiological and behavioral experiments to better understand the functions of different sensilla and mechanisms related to semiochemically based pest control strategies.

## 1. Introduction

Global trade networks in combination with other factors, including the climate change, play an important role in the distribution of invasive insects in various parts of the World [1,2]. Beetles of the family Buprestidae, jewel-beetles, comprise some economically damaging invasive pests which have recently become established in North America, Europe and Asia [3,4,5,6].

The Cypress jewel beetle or Cypress borer, *Lamprodila* (*Palmar*) *festiva festiva* (Linnaeus, 1767) (Chrysochroinae: Poecilonotini) (hereafter referred only as *L*. *festiva*), is a new invasive species native to the Mediterranean region, including southern Europe, which has recently expanded its range to central and eastern Europe, and to the Russian Black Sea coast [7,8,9,10]. In natural conditions, larvae of this species feed on native junipers (*Juniperus* spp.), cypresses (*Cupressus* spp.) and sandarac (*Tetraclinis articulata* (Vahl) Masters, 1892) [8]. However, especially in the areas of invasion, it has adapted to various ornamental, usually introduced, Cupressaceae, including different species and cultivars of *Callitris* Ventenat, 1808, *Chamaecyparis* Spach, 1841, *Platycladus* (Linnaeus) Franco, 1949, *Thuja* Linnaeus, 1753, and some hybrids. Hence, *L. festiva* has become a dangerous pest in urban landscapes and nurseries in, e.g., Austria [11], Czech Republic [12], Germany [13], Hungary [14,15], Luxembourg [16], Romania [7,9,17], Slovakia [18], Slovenia [19], and Russia [8]. The speed of the *L. festiva* invasion was documented in the Hungarian case. This species was first found in the southern part of the country in 1999 [20], and for many years, this was the only known locality for *L. festiva* in Hungary [21]. Therefore, this beetle became protected by the law of the Hungarian Ministry of Environment and Water (18/2008, 19.VI.), with a natural conservation value for each specimen set to 50,000 HUF. In 2012, amateur beetle collectors reported several specimens from Budapest, and one year later, a huge swarming of *L. festiva*, mostly on *Platycladus occidentalis* (L.) Franco, 1949, was observed in more districts of the capital [14]. Specimens were probably imported with infested trees. Since this species quickly became a serious pest of ornamental Cupressaceae in the capital, it was deleted from the list of protected beetle species in Hungary by the law of the Hungarian Ministry of Agriculture (66/2015, 26.X.).

Pest control and management is fundamental for the reduction in the damage caused by invasive pest species, including jewel-beetles. This often includes the development of biological control programs [22], but also the use of the pheromone traps [23,24]. The antennal sensilla are specialized structures which play an important role in gustation, mechano-, hygro-, thermo- and chemoreception [25,26]. The sensilla with an olfactory function detect sex pheromones and host plant volatiles, and therefore, it is important to study their ultrastructure and distribution. However, despite some recent progress, our knowledge of olfactory processes, chemoreception, and antennal sensory systems in Buprestidae is still limited [24,27,28,29,30,31]. In the most comprehensive study, Volkovitsh [28] examined antennal micromorphology for 412 jewel-beetle species classified in 316 genera, and identified the main sensillum types and subtypes present in Buprestidae. Recent studies even suggested that some closely related species may substantially differ in the antennal sensillar equipment [29,30], and therefore, the study of the antennal receptors of each individual pest species is needed.

In this study, we examined male and female antennal micromorphology of *L. festiva* by means of the scanning electron microscopy. Our main goals were to identify the sensillum types, discuss their possible functions, and compare their morphology, number, and distribution with the sensilla of other Buprestidae. The detailed study of the antennal sensillar equipment of *L. festiva* should serve as background information for subsequent chemical ecology research focused mainly on the olfactory sensory system.

## 2. Materials and Methods

**Figure 1:**
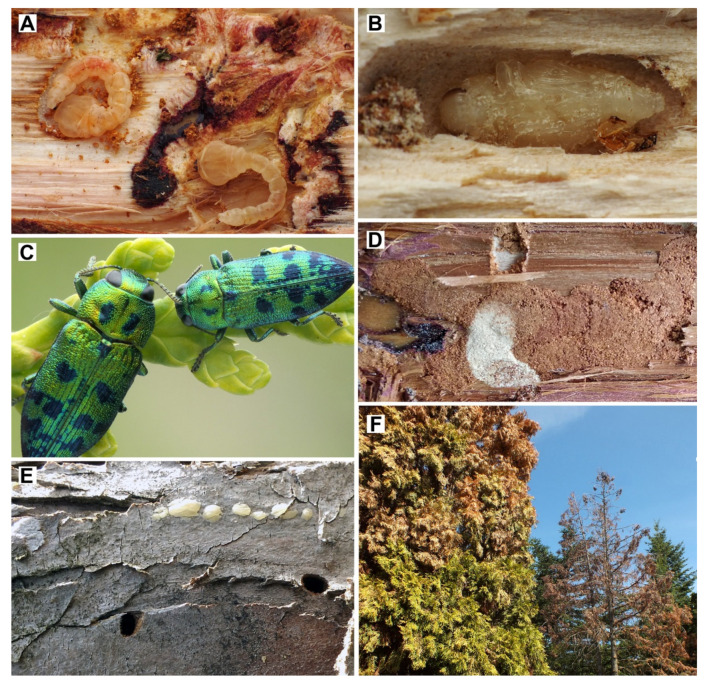
*Lamprodila festiva*. (**A**) Larvae under the bark of *Platycladus occidentalis*; (**B**) pupa; (**C**) adult specimens; (**D**) larval tunnels under the bark; (**E**) exit holes on branches; (**F**) trees damaged by *L. festiva* in Hungary.

We examined the morphology, number, and distribution of the sensilla in both male and female antennae of *Lamprodila festiva*. For each sex, we examined both the left and right antennae of three specimens (body length: males: 9.5–9.6 mm, females: 6.9–7.1 mm). All individuals were collected by the second author and bear the following label data: “Hungary, Pest county, Nagymaros, Rigó-hegy, from garden, reared from *Platycladus occidentalis* (L.) Franco, 1949, 01.–11.06.2019., leg. T. Németh” (Figure 1A–F). The specimens are deposited in the collection of the first author (Nantes, France). Additional specimens from the same collecting event are deposited in the Hungarian Natural History Museum (Budapest, Hungary). For the scanning electron microscopy (SEM) examination, the antennae were cleaned in acetone, dehydrated in 100% ethanol, air-dried, and mounted either on its ventral or dorsal face on a specimen holder. After coating with gold and palladium, antennae were examined using a Jeol J.S.M. 6400F scanning electron microscope (Jeol Ltd., Tokyo, Japan) at 12 kV. The numbers of each sensillum type were calculated from the counts of both faces of 11 antennomeres of the antenna with the SEM. We measured the lengths and basal and distal width of the different sensilla present on the antennae during the acquisition (10 measurements per sensillum type). Only sensilla that could be entirely viewed perpendicularly were measured for length to ensure the accuracy of the presented data. Sensillum terminology and classification follows Zacharuk [32], Faucheux [26], Yi et al. [30] and Faucheux et al. [33]. For the (sub)apical sensory fields on antennomeres IV–XI, in which most sensilla are located, we use the term “apical depressions” [30]. The classification and taxonomy of *Lamprodila* follows Kubáň [34] and most subsequent authors [8,9,10,15,17,18]; but see [7,11,35,36].

## 3. Results

### 3.1. Gross Morphology of Antennae in L. festiva

**Figure 2:**
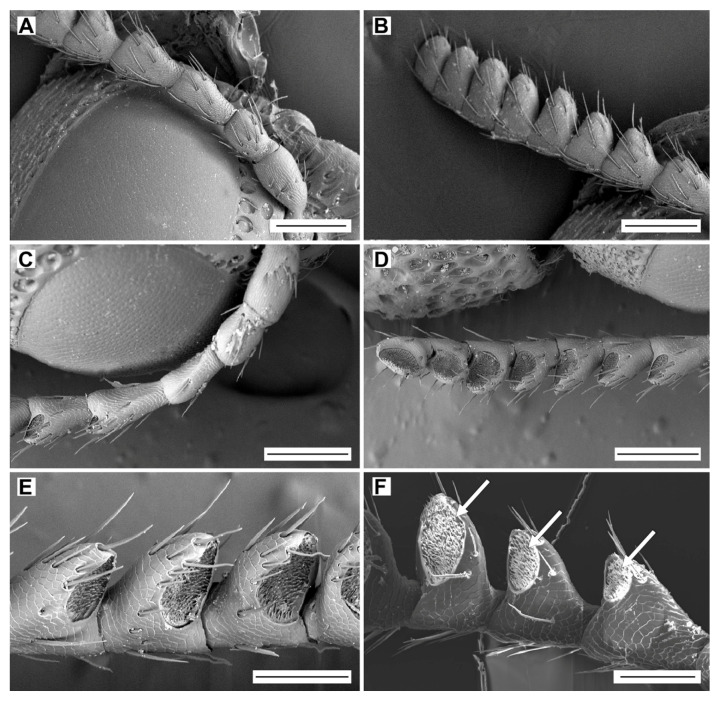
General morphology of antennae of *Lamprodila festiva*. Female, left antenna: (**A**) antennomeres I–VI, dorsal view; (**B**) antennomeres IV–XI, dorsal view; (**C**) antennomeres I–V, ventral view; (**D**) antennomeres V–XI, ventral view; (**E**) antennomeres VI–IX, details of apical depressions, ventral view. Male, right antenna: (**F**) antennomeres IV–VI, details of apical depressions (arrows), ventral view. Scale bars = (**A**–**D**): 200 µm; (**E**,**F**): 100 µm.

The shape and structure of antennae are similar in both sexes (Figure 2, Table 1). The antenna of *L. festiva* is rather short, serrate, and consists of 11 antennomeres: a scape (antennomere I), a pedicel (antennomere II), and a flagellum composed of nine flagellomeres (antennomeres III–XI) (Figure 2A–D). In the cross section, the antennomeres I–III are circular, and the antennomeres IV–XI are flattened (IV–IX subtriangular, X–XI suboval). The antennomeres I–III are only slightly wider distally than basally, while the antennomeres IV–XI are gradually widened towards the distal part, and they are notably wider towards the apex (Table 1). The antennal microsculpture on both faces consists of cuticular scales. On the antennomeres IV–XI (i.e., all flagellomeres except the first one), most sensilla are located in the apical depressions on the ventral faces (Figure 2D–F).

### 3.2. Types of Antennal Sensilla in L. festiva

We identified four main types of antennal sensilla in both sexes of *L. festiva*, including the sensilla chaetica (seven subtypes), sensilla basiconica (five subtypes), multiporous grooved pegs (MGP1 and a MGP2 complex), and Böhm sensilla. Their morphological characteristics are summarized in Table 2. The numbers of different types and subtypes of sensilla for each antennomere and in total are given in Table 3, Table 4, Table 5 and Table 6. Except for Böhm sensilla, which are located on the antennomeres I–II, and sensilla chaetica, which are distributed on both faces of all antennomeres, all other sensilla are located in the apical depressions on the ventral faces of the antennomeres IV–XI (i.e., all flagellomeres except for the first one). The dimensions of the apical depressions on the flagellomeres in males and females are given in Table 4.

### 3.3. Sensilla Chaetica (C1–C7)

**Figure 3:**
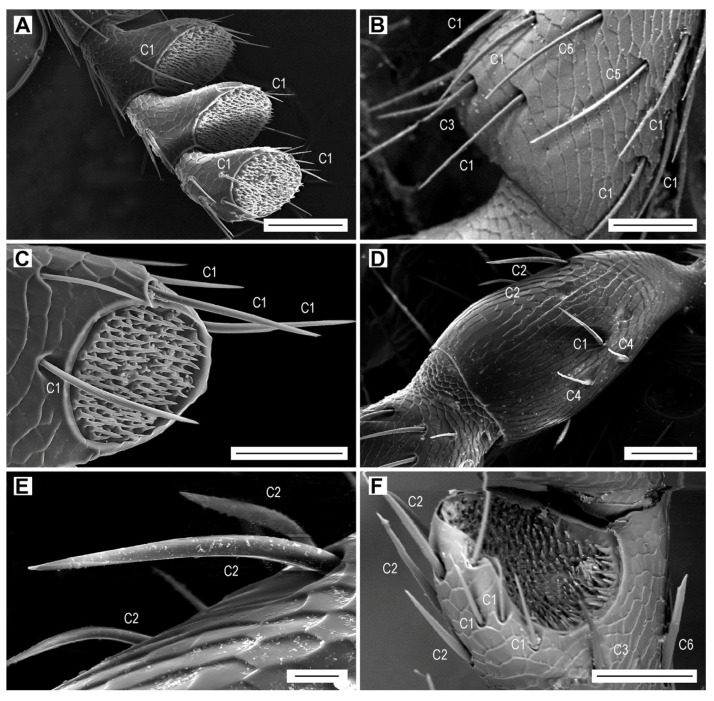
Aporous sensilla chaetica C1 and C2. (**A**) Antennomeres IX–XI with sensilla C1, ventrolateral view; (**B**) antennomere IV with sensilla C1, C3 and C5, dorsal view; (**C**) antennomere XI with sensilla C1, ventral view; (**D**) antennomere I with sensilla C1, C2 and C4, dorsal view; (**E**) sensilla C2 on antennomere I; (**F**) sensilla C1, C2, C3 and C6 on antennomere VII, ventral view. Scale bars = (**A**): 100 µm; (**B**–**D**,**F**): 50 µm; (**E**): 10 µm.

The sensilla chaetica are long hairs which are more or less evenly distributed on all antennomeres (Figure 3A,B, Table 3). They are the only type of sensilla on the dorsal face of the antenna. On the ventral face, they are always located outside of the apical depressions which contain different sensillum types. The sensilla chaetica form 12.1% of the total antennal sensilla in males and 15.0% in females (Table 6). We distinguished seven subtypes of sensilla chaetica (hereafter referred to as C1–C7) on the antennae of *L. festiva*.

Sensilla C1 are by far the most numerous sensilla chaetica (Figure 3A,B). They are inserted in a relatively wide cuticular cavity. These sensilla are slender, with a diameter which decreases gradually from the base to the sharp tip, and with a wall striated longitudinally by 15 shallow furrows. We have not found any wall pores or terminal pore (Table 2). These sensilla are quite variable in length which generally increases towards the antennal apex. The sensilla C1 on the scape are 22–40 µm long, whereas those on the distal antennomeres might be longer than 100 µm. In addition, the length of sensilla C1 within the same antennomere differs; for example, the sensilla grouped at the edge of the apical depression vary in length, usually from 45 to 83 μm, and the single isolated sensillum in the middle of the antennomere is approximately 95 µm long (Figure 3A). These sensilla have a specific localization on the antennomeres, particularly on the flagellomeres. They are the most numerous in the distal part of each antennomere, where they surround the apical depression (Figure 3C). Sensilla C1 are the only sensilla present on the inner edge of flagellomeres, and they are together with the sensilla C2 on the outer edge (Figure 3A). Their number per flagellomere is relatively constant (Table 3). In both sexes, they are more numerous on the dorsal face, but the female antenna possesses more sensilla (Table 3). There are also differences between sexes in the length of sensilla C1 on dorsal and ventral faces, for example on the median antennomeres VII–VIII. In females, the sensillum lengths on each particular face are quite homogenous, but the lengths differ considerably between faces; 90–100 µm on the dorsal face, and 35–45 µm on the ventral face. In males, the sensilla C1 are 65–80 µm long on the dorsal face and 35–65 µm long on the ventral face.

Sensilla chaetica C2 are usually curved and saber-shaped (Figure 3D–F). They are on average shorter and basally wider than sensilla C1 (Table 2). Their wall has a dozen longitudinal furrows and is not perforated by any sensory pore. They are inserted into a large funnel-shaped depression. In most antennomeres, their length is almost homogenous and varies from 55 to 65 µm (Figure 3F). However, on the scape, they are 30–70 µm long (Figure 3D,E). They are usually located on the inner edge of the antennomeres, but occasionally they are also on the ventral face and the outer edge (Figure 2E and Figure 3F). They are missing on the dorsal face of the antenna, which is equipped only with sensilla C1 (Figure 3B). There are only up to five sensilla C2 per flagellomere.

**Figure 4:**
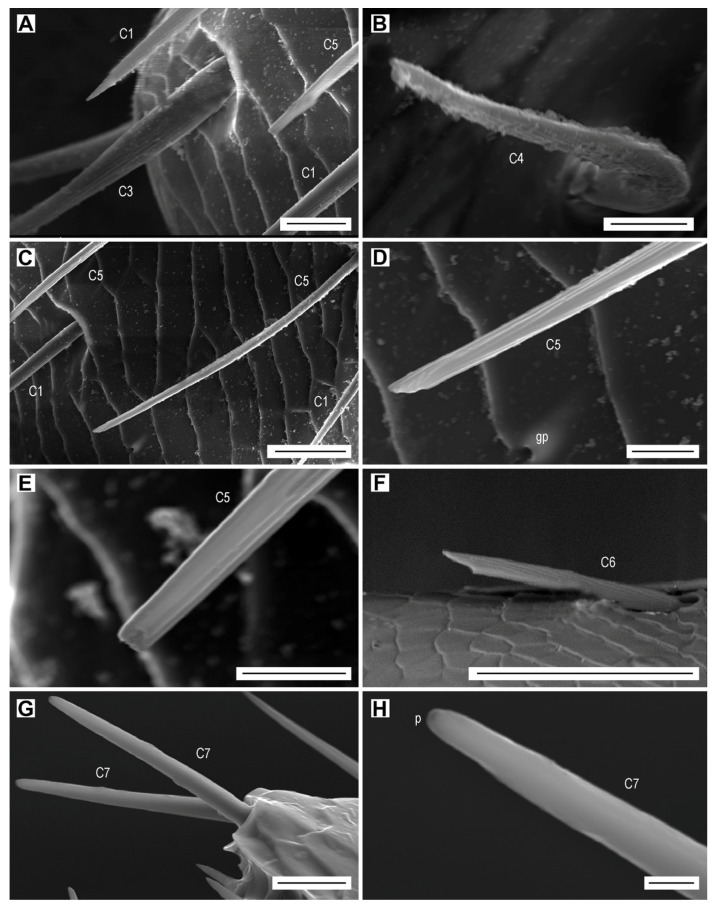
Sensilla chaetica. Aporous sensilla chaetica C3 and C4: (**A**) sensillum C3 on antennomere IV; (**B**) sensillum C4 on antennomere I. Uniporous sensilla chaetica C5–C7: (**C**) sensilla C5 on antennomere IV, dorsal view; (**D**) apex of sensillum C5 on antennomere IV; (**E**) apex of sensillum C5 on antennomere IV; (**F**) sensillum C6 on antennomere VII; (**G**) sensilla C7 on antennomere X; (**H**) apex of sensillum C7 showing the terminal pore (p). Scale bars = (**A**,**G**): 10 µm; (**B**,**D**,**E**): 5 µm; (**C**): 20 µm; (**F**): 50 µm; (**H**): 2 µm.

Sensilla chaetica C3 are somewhat intermediate between sensilla C1 and C2. They are similar in length to sensilla C1 (Table 2), and usually they have a proximal third of their length identical to a sensillum C2, and the distal two thirds comparable to a sensillum C1 (Figure 3B and Figure 4A). They are inserted in a relatively wide cuticular cavity (Figure 4A). Similarly to sensilla C1 and C2, they do not have the sensory pores. We found only two to four sensilla C3 on the antennae of three females and two males.

Sensilla chaetica C4 occur only on the scape and pedicel (Figure 3D). They are the shortest among all subtypes of sensilla chaetica (Table 2). They are bent just above their insertion into the antennomere surface, and have a smooth surface and a relatively sharp tip, both without sensory pores (Figure 4B). These sensilla are found on the outer edge of the antennomeres, two on the scape and two on the pedicel.

Sensilla chaetica C5 are present only in females. Two of them are on the dorsal face of each of the antennomeres IV–XI, located in the middle of the distal part of antennomere (Figure 3B). They are subparallel to each other but because they are slightly curved, they face each other by their concave parts (Figure 3B and Figure 4C). The wall includes 10 longitudinal furrows, which are deeper than those in the previous subtypes of sensilla chaetica. They are among the longest and slenderest sesilla chaetica (Table 2), and can be easily recognized by their subparallel sides, with basal and distal diameters of approximately 2.9 and 2.5 µm, respectively. Additionally, they have a special apex, which is more or less obliquely truncated (Figure 4D), most probably with a terminal pore (Figure 4E).

Sensilla chaetica C6 were found only on the outer edge of a single antennomere (VII or VIII) in two females (Figure 3F). They are relatively short and stout, similar to sensilla C2, and their wall includes 10 furrows (Table 2). This sensillum has subparallel sides for almost its whole length but in the subdistal region it is abruptly narrowed to form two tips—one subapical and one apical (Figure 4F). This particular apical portion of the sensillum suggests the existence of a terminal pore.

**Figure 5:**
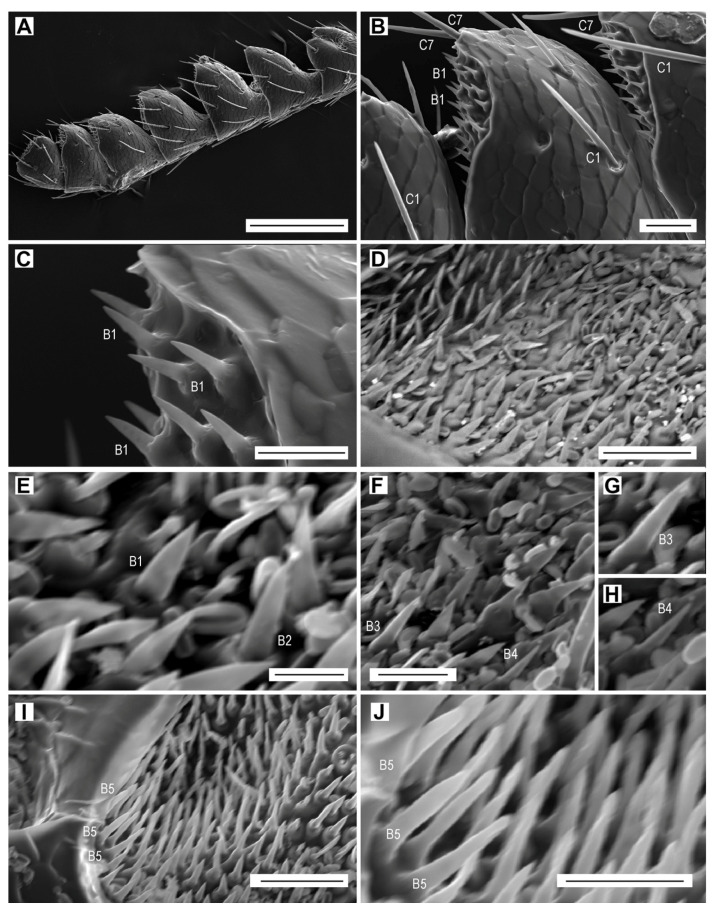
Sensilla basiconica. (**A**) Male antenna, dorsal face; (**B**) antennomere X with sensilla basiconica B1 in the apical depression, and sensilla chaetica C1 and C7; (**C**) sensilla basiconica B1; (**D**) apical depression on antennomere X, showing the field of sensilla basiconica; (**E**) sensilla basiconica B1 and B2; (**F**) sensilla basiconica B3 and B4; (**G**) sensillum basiconicum B3; (**H**) sensillum basiconicum B4; (**I**) sensilla basiconica B5; (**J**) close-up of sensilla basiconica B5. Scale bars = (**A**): 200 µm; (**B**,**D**,**I**): 20 µm; (**C**,**F**,**J**): 10 µm; (**E**): 5 µm; (**G**,**H**): not to scale.

Sensilla chaetica C7 are 15–33 µm long, so they are relatively short compared to the remaining sensilla chaetica (Table 2). They are parallel-sided, with walls equipped with 15 more or less faint striae, and with a terminal pore (Figure 4G,H). These sensilla are located at the upper outer corner of the apical depressions of each of the antennomeres IV–XI in both male and female (Figure 5B). In females, there is a single sensillum on each of the antennomeres, whereas in males, there are usually two sensilla near to each other on each antennomere.

### 3.4. Sensilla Basiconica (B1–B5)

All five subtypes of sensilla basiconica are present in large numbers inside the apical depressions of the antennomeres IV–XI. The number of sensilla basiconica per apical depression usually depends on the size of the depression, and varies from 72 to 198 in males and from 63 to 181 in females (Table 4). The total number of sensilla basiconica in *L. festiva* is larger in males than in females; they form 80.9% of the total antennal sensilla in males and 76.3% in females (Table 6). Individual subtypes are distinguished based on their general aspect, size, basal diameter, and location within the depressions (Table 2). Sensilla B1 are located mainly at the distal edge of depressions (Figure 5A–C), but they are also in other places within depressions, mixed with other subtypes of sensilla basiconica (Figure 5D,E). They are smooth, sharp-tipped, subparallel-sided or only gradually narrowed until about mid-length and then more or less abruptly narrowed towards the apex. Sensilla B2 are slightly shorter, slenderer, and apparently less pointed than sensilla B1, near which they are usually located (Figure 5E). Sensilla B3 are longer than the previous subtypes, usually gradually narrowed towards the apex, with a sharp pointed tip (Figure 5F,G). They are located close to the external edge of depressions. Sensilla B4 are the narrowest and sharpest among sensilla basiconica (Figure 5F,H). Sensilla B5 are the longest sensilla basiconica (Table 2). They are located in high numbers near the lower internal edge of the depression (Figure 5I,J). In all subtypes of sensilla basiconica, wall pores are hypothesized but not clearly visible using the SEM.

### 3.5. Multiporous Grooved Pegs (MGP1, MGP2)

**Figure 6:**
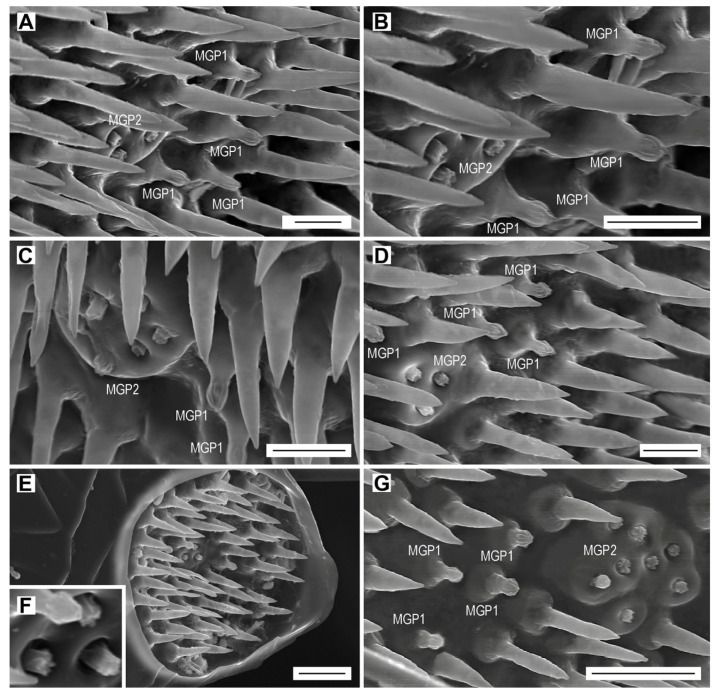
Multiporous grooved pegs MGP1 and MGP2 (complex). (**A**) Sensilla MGP1 and a complex of four sensilla MGP2 on female antennomere VI; (**B**) close-up of sensilla MGP1 and MGP2; (**C**) Sensilla MGP1 and a complex of six sensilla MGP2 on female antennomere VIII; (**D**) sensilla MGP1 and a complex of three sensilla MGP2 on female antennomere V; (**E**) position of sensilla MGP1 and a complex of three sensilla MGP2 within the apical depression of female antennomere IV; (**F**) close-up of sensillum MGP1 and a complex of three sensilla MGP2; (**G**) sensilla MGP1 and a complex of eight sensilla MGP2 on female antennomere XI. Scale bars = (**A**–**D**): 5 µm; (**E**,**G**): 10 µm; (**F**): not to scale.

The individual multiporous grooved pegs are called “subtype 1” (MGP1), whereas the sensory complex formed by a cluster of modified multiporous grooved pegs located on a thick plate is called the “MGP2 complex”. The MGP1 is a cone-shaped sensillum with a bulbous head. It is smooth at the base and has six to eight deep and relatively wide grooves running from about the middle to apex (Figure 6A–G). These grooves are in between the finger-like ribs which run from the tip of the peg. Some multiporous grooved pegs in *L. festiva* are stout and relatively short, with the sensory cone reaching only up to 3.8 µm in length, while some are more elongated, with a cone of about 5.0 µm long. They are inserted into a prominent bulbous base, which has an outer diameter up to 3.7 µm and height up to 2.5 µm (Figure 6A–G). These sensilla can be found in the apical depressions of each of the antennomeres IV–XI, usually located around the middle of the depression. They are either separate or form small clusters (Figure 6A–G). There were no significant differences between males and females in the abundances of this type of sensilla (Table 5). Their number per antennomere increases from antennomere IV to antennomere XI, and varies from three to six in males and from three to five in females (Table 5). They form 2.4% of the total antennal sensilla in males and 2.6% in females (Table 6).

The MGP2 sensory complex is a special cluster of several sensilla inserted into a thick circular plate (Figure 6A–G). A single MGP2 complex is present in the middle of each apical depression on the antennomeres IV–XI. Each sensillum in the MGP2 resembles the more or less modified distal half of a multiporous grooved peg of a subtype 1 (Figure 6A–G). Many sensilla are cylindrical, with wall ribs mainly apparent apically, and forming a cavity on the tip. Most sensilla of the MGP2 have the following lengths: 0.75, 1.1, and 1.6 µm. The shortest sensilla are sunken in the plate whose diameter varies according to the antennomere, e.g., 7.9 µm for the antennomere V (Figure 3C and Figure 6D), 8.5 µm for the antennomere VIII (Figure 6C), and 13 µm for the antennomere XI (Figure 6G). The circular plate is usually smooth but that on the terminal antennomere has a rough surface (Figure 6G). The number of sensilla per complex/antennomere increases from antennomere IV to antennomere XI, and varies from one to four in males and from three to eight in females. Therefore, the total number of these sensilla in females is almost two times higher than in males (Table 5), and they form 1.6% of the total antennal sensilla in males and 3.3% in females (Table 6).

### 3.6. Böhm Sensilla

**Figure 7:**
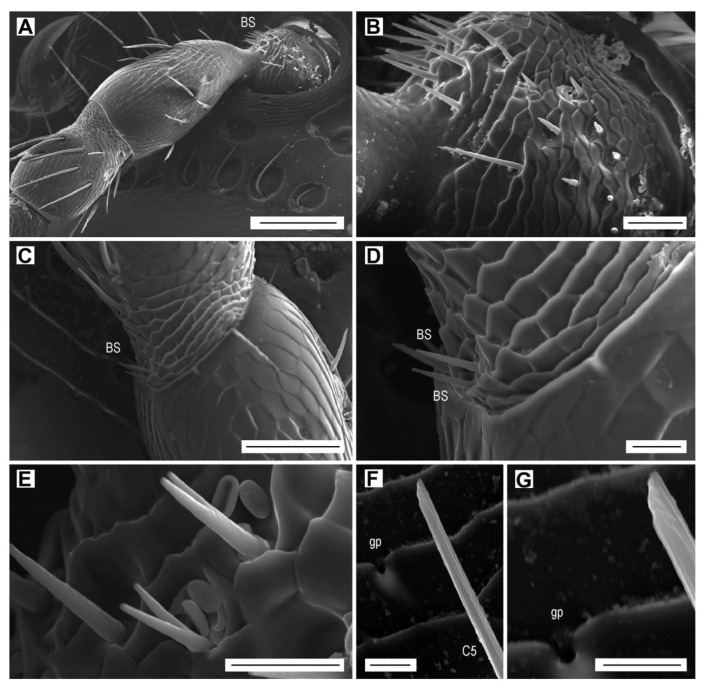
Böhm sensilla (BS) and glandular pore (gp). Böhm sensilla: (**A**) Scape and pedicel of the left female antenna, dorsal face; (**B**) Böhm sensilla on the scape; (**C**) Böhm sensilla at the base of pedicel; (**D**) close-up of Böhm sensilla; (**E**) bifurcated Böhm sensilla on the scape. Glandular pore: (**F**) glandular pore near sensillum C5 on antennomere IV; (**G**) close-up of glandular pore. Scale bars = (**A**): 100 µm; (**B**): 20 µm; (**C**): 50 µm; (**D**,**E**): 10 µm; (**F**,**G**): 5 µm.

Böhm sensilla are short, smooth, thorn-like bristles with sharp tips (Figure 7A,B). They are located in three clusters at the base (condyle) of the scape (Figure 7A,B), and in one inner lateral cluster at the base of pedicel (Figure 7C,D). Their lengths vary from 17.3 to 23.5 µm within the inner lateral cluster on scape, 5.2 to 12.7 µm within the ventral cluster on scape (Figure 7B), or remains uniform, 13 µm, within the cluster on pedicel (Figure 7D). Several sensilla on the scape are bifurcate, with the bifurcation beginning either at the base or at the distal half (Figure 7E). There are about 40 Böhm sensilla located on the ventral side of the scape, and only four sensilla on the pedicel. These sensilla form 2.9% of the total antennal sensilla in males and 2.7% in females (Table 6).

### 3.7. Glandular Pores

Exocrine glandular pores, 0.9 µm in diameter, were found among the sensilla (but never associated with sensilla) at the distal edge of a cuticular scale (Figure 4D and Figure 7F,G). There are no more than two pores per antennomere.

## 4. Discussion

In this paper, we focused on the antennal micromorphology in both sexes of *L. festiva* in order to investigate the typology, location, number, and possible functions of antennal sensilla. We identified four main types of antennal sensilla based on morphology, i.e., sensilla chaetica, which were further divided into seven subtypes, sensilla basiconica of five subtypes, multiporous grooved pegs of two subtypes, and Böhm sensilla (Figure 3, Figure 4, Figure 5, Figure 6 and Figure 7, Table 2, Table 3, Table 4, Table 5 and Table 6). Volkovitsh [28], who studied antennae of 412 buprestid species belonging to 316 genera, also identified several subtypes of chaetoid (=chaetica), basiconic (=basiconica) and multiporous basiconic grooved-wall (=MGP) sensilla within the family. Additionally, he found in Buprestidae several types of sensilla which were not found in *L. festiva*, such as campaniform sensilla or specialized male sensilla. Buprestidae are a species-rich family with approximately 15,000 described species [37], and consequently, their sensillar equipment is also diverse. Some studies suggested that even species within the same genus may considerably differ in the types, distribution, numbers, and function of antennal sensilla [29,30]. Most sensilla are located in large (sub)apical sensory fields on the ventral faces of the antennomeres IV–XI. These fields of sensilla were proved to be of a taxonomic importance [28]. They are variously named in the literature, e.g., fossae [28], apical depressions [29,30], or sensorial pits [31]. Similarly, the sensillum nomenclature is very often inconsistently used in various groups of insects; however, it should be noted that the situation in Buprestidae is much better than in some other groups [33,38]. We discuss the antennal sensillum types in *L. festiva* as well as their alternative names used by different authors and identical/similar sensilla in other insect lineages in the following sections. The use of consistent nomenclature for antennal sensillum types within the given group is necessary for the reliable identification of certain sensillum (sub)types and comparisons between related taxa.

### 4.1. Sensilla Chaetica

Sensilla chaetica C1–C4 of *L. festiva* have longitudinal furrows and do not possess a terminal pore or wall pores. Contrary to the sensilla in apical depressions, they are well represented on both faces of antennomeres. They are typical aporous sensilla chaetica which are present in all Coleoptera. Several subtypes of sensilla chaetica with similar distribution on antennae were identified in *Agrilus mali* [30], and sensilla C1 are identical to sensilla chaetica of *Agrilus planipennis* [29]. The cross section of these sensilla observed by the use of transmission electron microscopy shows that they have a non-perforated wall and a single sensory neuron with the dendrite terminating in the form of a tubular body [29]. These ultrastructural characteristics suggest that these sensilla are typical tactile mechanoreceptors which respond to mechanical stimuli [32,39]. Crook et al. ([29]; p. 1105) hypothesized that “the longer sensilla (chaetica) are located on the scape and may regulate movement/rotation of the entire antenna”. However, the movements, rotation and position of insect antenna (including Buprestidae) are controlled by Böhm sensilla, which were not examined in the above-mentioned study [29].

Sensilla C5 and especially C6, which were found exclusively on the female antenna, are identical to sensilla chaetica C4 present on the labial palpus of *Dipseudopsis oliveri* Oláh and Johanson, 2010 (Trichoptera) [40]. Their apical part is notably asymmetrical, with the distal tip being very sharp and without a pore, and the subdistal tip being blunt, sometimes reduced in size or even forming only a small protuberance with a visible terminal pore. These sensilla are superficially similar in the general shape to sensilla trichodea Tr2 and Tr3 of *Agrilus mali* [30]; however, the latter two sensilla differ considerably from sensilla C5 and C6 in the smooth wall which is perforated by pores. We suggest that both sensilla C5 and C6 of *L. festiva* could be gustatory receptors. The relatively rare uniporous sensilla chaetica C7 of *L. festiva* are also gustatory receptors. In other Buprestidae, gustatory sensilla with similar location and numbers include sensilla U1 (various genera and species) [28] or sensilla basiconica Ba4 (*A. mali*) [30]. The numerous uniporous sensilla (U) in *A. planipennis* also bear the gustatory function [29]. Some buprestid species show the sexual dimorphism in the number of gustatory uniporous sensilla [29], but other species do not [30]. Males of *L. festiva* possess more uniporous sensilla chaetica C7 than females, but on the other hand, only females possess sensilla C5 and C6, for which we also hypothesize gustatory function. Various authors suggested that the contact chemical cues are important for mate recognition in Buprestidae, particularly by males, rather than using pheromones at any distance [29,41]. The antennal contact most probably plays an important role for mate recognition also in *L. festiva*. This is supported by the fact that we failed to find the smooth-walled multiporous sensilla trichodea, which are sensitive to sex pheromones and host plant volatiles [42]. Sensilla trichodea were reported for example in *A. mali* [30], but similar to in *L. festiva*, this sensillum type was absent in *A. planipennis*, in which the gustatory sensilla in males outnumber the sensilla of the same function in females [29].

### 4.2. Sensilla Basiconica

The sensilla basiconica B1 of *L. festiva* resemble multiporous sensilla basiconica Ba2 of *Agrilus mali* [30] and sensilla B5 of *Agriotes* spp. [33]. All sharp-tipped sensilla basiconica within the apical depressions in *L. festiva* most probably have an olfactory function. The depressions, with respect to the position of antenna during flight, may enable the concentration of odorous stimuli within the cavity when they are exposed directly to the airflow. Because the probability of an odor molecule striking a sensillum would increase, the efficiency of the odor detection by the sensilla basiconica would increase, too. Sensilla basiconica form 76% (female) to 81% (male) of all antennal sensilla, and therefore, the antennal apical depressions in *L. festiva* have a predominant olfactory function. The sensory fields within the apical depressions in *A. planipennis* are composed mainly of uniporous chemoreceptors [29]. Therefore, on the contrary to the situation in *L. festiva*, the depressions have an important contact chemoreceptive function. Most sensilla basiconica in *A. planipennis* are located on the distal part of flagellomeres, outside the apical depressions, and they most probably form the main olfactory apparatus for detecting host tree volatiles for both sexes of this species [29].

Sensilla basiconica Ba3 and Ba4 with a special apical part (i.e., a filament-like long tip, and a pore at the papillous tip), which are present in *A. mali* [30], have not been found either in *L. festiva* or in *A. planipennis* [29].

### 4.3. Multiporous Grooved Pegs

Based on their morphology, the multiporous grooved pegs (MGP) of *L. festiva* are probably identical to some sensilla basiconica in various Coleoptera, e.g., type 3 of *Leptura* spp. (Cerambycidae) [43], type 2 of *Callosobruchus rhodesianus* (Pic, 1902) (Chrysomelidae) [44], types 7 and 8 of Drilini (Elateridae) [45], and type 7 of *Agriotes* spp. (Elateridae) [33], but also to sensilla coeloconica of *Tetrigus lewisi* Candèze, 1873 (Elateridae) [46] or the grooved pegs of *Elater ferrugineus* (Linnaeus, 1758) [47]. In Buprestidae, these sensilla were named as multiporous basiconic groove-walled sensilla M5 [28] or multiporous grooved pegs [30]. They were not reported for *Agrilus planipennis* [29] and *Capnodis tenebrionis* [31]. Seada and Hamza [48] examined multiporous grooved pegs in the tenebrionid *Tribolium castaneum* (Herbst, 1797), and noted the presence of longitudinal narrow pores on the bulbous distal peg of these sensilla. The multiporous grooved pegs of beetles are called sensilla coeloconica in Lepidoptera, and are classified as double-walled multiporous sensilla [39]. Ultrastructural observations of sensilla coeloconica indicated that odorous molecules enter the sensillum lymph lumen through the longitudinal grooves along the peg [49]. Based on electrophysiological studies, the main function of these sensilla might be olfactory [50,51,52]. While the response spectrum of sensilla basiconica and auricillica in Lepidoptera is diverse and includes, e.g., the plant volatiles and sex pheromone components [53,54], the neurons housed in sensilla coeloconica are stereotypically tuned to acids, aliphatic aldehydes, and amines [51,54], as documented also in other insect orders, such as Odonata [55], Blattodea [56], and Diptera [57]. The presence of MGP in both sexes of *L. festiva* suggests that they perceive odors from host plants for feeding and oviposition.

The sensilla complex, which is formed by multiporous grooved peg sensilla MGP2, is an assembly of individual sensilla resembling short MGP1, which are grouped together in about the middle of each apical depression. Some other sensilla complexes were identified in insects, but these are usually represented by several fused sensilla which together form a single dome-shaped structure. These complexes include the multiporous basiconic sensillum complex (MBSC) [58,59] (=antennal sensory complex of Scott and Zacharuk [60], the large sensillum basiconicum on the antennae of beetle larvae [61], and the styliform complex sensilla on antennae of the sphingid moth *Manduca sexta* (Linnaeus, 1763) [62]. The MBSC represents the fusion of 12 individual sensilla into a compound chemoreceptor [61]. According to Roppel et al. [63], the function of atmospheric chemoreception of the sensory complex is suggested by the presence of channels in the thin cuticle, which communicate directly with the external environment. The high number of chemosensory neurons of this multiporous sensory organ suggests a possible ability of fine odor discrimination related to plant-host location [64]. The styliform complex sensilla of *Manduca sexta* is formed by several contiguous sensilla styloconica, the number of which depends on the location of the complex on the antenna [62]. Similarly, the number of individual sensilla, which form the MGP2 sensory complex in *L. festiva*, varies on antennomeres IV–XI (Figure 6). The complexes probably optimize the performance of individual sensilla by increasing their efficiency.

The sensory complex formed by sensilla MGP2 was not mentioned in the literature focused on the antennal sensilla of Buprestidae [28,30,31]. The antenna of *L. festiva* was not studied by Volkovitsh [28]; however, he examined some other species belonging to the same subfamily. On the low-magnification photographs of apical depressions on antennae of some Chrysochroinae, there are clearly visible small areas in the middle of each depression, which are without sensilla basiconica and hence might indicate the presence of the MGP2 complexes. This is especially visible in Figures 82 (for *Saundersina modesta* (Fabricius, 1781)) and 85 (for *Palmar* (*Scintillatrix*) *chinganensis* (Obenberger, 1940), the latter of which is currently a synonym of *Lamprodila suyfunensis* (Obenberger, 1934)). In the latter species, there is probably a single complex on antennomere X and two complexes on antennomere XI (Figure 85 in [28]). We believe the examination of the presence of sensilla MGP2 in other taxa of Chrysochroinae, especially various species of *Lamprodila*, would help us to understand the distribution of these structures within Buprestidae.

### 4.4. Böhm Sensilla

Böhm sensilla are mechanosensors which monitor the position and movements of the antenna in flight [25,26,65,66]. They are typically present on the first two antennomeres in various insect orders, including Coleoptera [26,67]. They are often difficult to observe, which might be the reason why this sensillum type was not mentioned in some previous studies focused on the antennal micromorphology of Buprestidae [27,29,31]. Similarly, Yi et al. [30] found Böhm sensilla only on the antennal condyle of scape in both sexes of *Agrilus mali*, but not on the base of the pedicel, where these sensilla usually form clusters [33,45]. In *L. festiva*, Böhm sensilla on the pedicel form a single cluster composed of four sensilla. Yi et al. [30] reported two subtypes of Böhm sensilla in *A. mali*; BB1, which are the usual Böhm sensilla, and BB2 with a bifurcate apex, which were rare and were found only in females. The latter are present also in *L. festiva* (Figure 7E). Similarly, bifurcate sensilla basiconica were recently reported for several lineages of Elateridae [33,68]. We hypothesize that the bifurcate sensilla, which are usually only rarely encountered on antennae, are only abnormally developed sensilla of various types.

## 5. Conclusions

*Lamprodila festiva* has relatively quickly become one of the most serious pests of ornamental Cupressaceae in Europe. However, our knowledge of the antennal sensory systems, intraspecific communication, as well as the insect–host chemical communication, has been limited. In order to overcome these limitations, we examined the variability in the sensory systems in *L. festiva* by the use of the scanning electron microscopy. Therefore, the present study should serve as a strong framework for more detailed research on the functions of the examined sensilla of this invasive pest species. In total, we identified 15 different (sub)types of sensilla, of which two are present only in females. More than three quarters of all sensilla were olfactory sensilla basiconica, which were housed in the apical depressions on antennomeres IV–XI. They represent the major antennal olfactory system and hence play an important role in the reception of environmental cues like host plant volatiles. We have not found any multiporous sensilla trichodea, which usually serve for detection of sex pheromones [42]. Therefore, we hypothesize that the antennal contact plays a role in mate recognition in *L. festiva*, as documented for some other Buprestidae [29,41].

Our study could also provide taxonomically important antennal morphological characteristics for future comprehensive systematic and phylogenetic research of Buprestidae. We suggest it would be especially interesting to examine the presence of the sensilla MGP2 complex within the jewel-beetle subfamily Chrysochroinae, and, more specifically, in all Poecilonotini and Dicercini, which are hypothesized to be sister-groups [10]. Antennal sensilla have already proved to be taxonomically important in various other insects [33,45,69,70], including Buprestidae [28].

## Figures and Tables

**Table 1:** Length, basal and distal width (mean ± SE) of each antennomere in both sexes of *L. festiva*, based on examination of both antennae for three specimens per sex. Anten., antennomere.

Anten.	Length (µm)		Width (µm)			
			Basal	Distal	Basal	Distal
	Male	Female	Male		Female	
I	244.5 ± 7.2	292.3 ± 8.5	61.9 ± 4.3	82.5 ± 4.3	62.7 ± 7.2	92.3 ± 5.4
II	125.8 ± 5.6	133.6 ± 6.2	82.7 ± 2.9	91.9 ± 3.2	83.4 ± 3.7	92.1 ± 4.6
III	142.5 ± 3.7	150.4 ± 3.5	62.3 ± 1.7	70.4 ± 2.9	62.8 ± 2.5	75.8 ± 4.3
IV	118.2 ± 3.2	187.0 ± 2.9	58.2 ± 3.3	145.4 ±4.1	62.0 ± 2.2	96.4 ± 6.4
V	161.0 ± 2.5	150.2 ± 3.7	58.7 ± 3.1	178.2 ± 3.6	75.3 ± 4.8	124.5 ± 3.9
VI	122.4 ± 3.8	116.5 ± 2.6	64.8 ± 2.8	181.8 ± 5.4	62.5 ± 5.6	121.0 ± 2.6
VII	129.0 ± 2.9	108.4 ± 2.1	64.4 ± 2.4	183.3 ± 4.9	62.3 ± 2.1	129.2 ± 7.6
VIII	122.5 ± 4.7	104.9 ± 6.3	63.5 ± 4.1	160.9 ± 3.7	71.2 ± 3.6	125.0 ± 2.3
IX	112.7 ± 3.5	108.3 ± 4.2	62.1 ± 3.1	161.3 ± 4.3	54.4 ± 5.2	133.6 ± 5.9
X	112.9 ± 2.9	96.4 ± 3.1	59.8 ± 3.9	154.8 ± 3.1	58.9 ± 4.8	112.7 ± 3.4
XI	103.2 ± 4.2	133.1 ± 5.8	59.3 ± 2.6	96.7 ± 3.4	50.3 ± 3.7	96.6 ± 2.9
Total	1494.7 ± 14.9	1561.3 ± 18.5	-	-	-	-

**Table 2:** Morphological characteristics of different sensillum types and subtypes on the antenna of *L. festiva*, based on examination of 20 measurements per sensillum type (10 measurements per sex). ?, pores hypothesized but not clearly visible in the studied material; MGP, multiporous grooved pegs.

Sensillum Type	Length (µm)	Basal Width (µm)	Pores
Chaeticum C1	82.3 ± 3.9	3.1 ± 0.2	no pore
Chaeticum C2	54.6 ± 2.7	4.8 ± 0.5	no pore
Chaeticum C3	93.8 ± 2.4	6.1 ± 0.3	no pore
Chaeticum C4	30.2 ± 0.3	2.7 ± 0.2	no pore
Chaeticum C5	92.5 ± 3.6	2.9 ± 0.1	terminal pore?
Chaeticum C6	60.7 ± 0.4	5.2 ± 0.3	terminal pore?
Chaeticum C7	29.8 ± 0.7	3.3 ± 0.4	terminal pore
Basiconicum B1	8.9 ± 0.5	2.2 ± 0.2	wall pores?
Basiconicum B2	7.4 ± 0.3	2.1 ± 0.3	wall pores?
Basiconicum B3	11.0 ± 1.4	2.1 ± 0.2	wall pores?
Basiconicum B4	8.2 ± 1.2	1.3 ± 0.2	wall pores?
Basiconicum B5	12.5 ± 1.0	2.0 ± 0.3	wall pores?
MGP1	4.2 ± 0.4	2.1 ± 0.2	wall pores?
MGP2	1.1 ± 0.2	1.0 ± 0.1	wall pores?
Böhm sensillum	18.3 ± 1.7	2.2 ± 0.1	no pore

**Table 3:** Average numbers of sensilla chaetica (C1–C7) per antennomere in both sexes of *L. festiva*, based on examination of both antennae for three specimens per sex. DF, dorsal face; LF, lateral faces; VF, ventral face.

	Male				Female			
Antennomere	DF	VF	LF	Total	DF	VF	LF	Total
I	5	5	6	16	5	6	7	18
II	6	4	5	15	8	5	6	19
III	7	2	2	11	6	2	5	13
IV	7	3	6	16	9	6	8	23
V	5	4	8	17	11	6	8	25
VI	4	5	6	15	7	5	5	17
VII	6	4	8	18	8	8	5	21
VIII	8	5	10	23	7	7	6	20
IX	7	6	6	19	6	5	7	18
X	3	4	7	14	3	4	4	11
XI	5	3	8	16	5	4	7	16
Total	63	45	72	180	75	58	68	201

**Table 4:** Length and greatest width (mean ± SE) of the antennal apical depressions (one each for antennomeres IV–XI), and number of sensilla basiconica B1–B5 in both sexes of *L. festiva*, based on examination of one specimen per sex.

Antennomere	Length (µm)		Maximum Width (µm)		Nr. of S. Basiconica	
	Male	Female	Male	Female	Male	Female
IV	81.8 ± 3.2	34.0 ± 2.4	41.3 ± 2.8	13.6 ± 2.7	72	63
V	118.1 ± 3.7	70.8 ± 2.9	62.3 ± 3.2	29.1 ± 4.1	95	81
VI	127.3 ± 2.3	75.0 ± 3.1	63.7 ± 2.5	33.3 ± 3.5	139	90
VII	121.5 ± 1.9	95.8 ± 2.6	78.2 ± 2.3	45.8 ± 3.6	162	124
VIII	133.7 ± 2.4	96.5 ± 4.2	81.4 ± 3.1	50.0 ± 2.3	175	158
IX	138.9 ± 4.5	104.2 ± 3.7	86.5 ± 5.7	66.5 ± 5.7	186	173
X	125.4 ± 3.8	70.8 ± 2.8	87.8 ± 4.9	67.2 ± 4.9	198	181
XI	136.3 ± 2.9	104.7 ± 4.5	95.2 ± 3.2	45.2 ± 3.2	177	149
IV–XI					1204	1019

**Table 5:** Numbers of multiporous grooved pegs 1 (MGP1) and sensilla of multiporous grooved peg complex (MGP2) in apical depressions of antennomeres IV–XI in both sexes of *L. festiva*, based on examination of both antennae for three specimens per sex.

Sex	Sensilla	IV	V	VI	VII	VIII	IX	X	XI	Total
Male	MGP1	3	3	3	4	6	5	6	6	36
	MGP2	1	2	3	3	3	4	4	4	24
Female	MGP1	3	4	4	4	4	5	5	5	34
	MGP2	3	3	4	5	6	7	7	8	43

**Table 6:** Numbers and percentages of the types of antennal sensilla in both sexes of *L. festiva*, based on examination of one specimen per sex.

Sensillum Type	Male		Female	
Chaetica C1–C7	180	12.1%	201	15.0%
Basiconica B1–B5	1204	80.9%	1019	76.3%
Multiporous grooved pegs 1	36	2.4%	34	2.6%
Multiporous grooved pegs 2	24	1.6%	43	3.3%
Böhm sensilla	44	2.9%	44	2.7%
All sensilla	1488		1341	
